# Development of genomic resources for Rhodes grass (*Chloris gayana*), draft genome and annotated variant discovery

**DOI:** 10.3389/fpls.2023.1239290

**Published:** 2023-09-04

**Authors:** Kellie Maybery-Reupert, Daniel Isenegger, Matthew Hayden, Noel Cogan

**Affiliations:** ^1^ Agriculture Victoria Research, AgriBio, The Centre for AgriBioscience, Bundoora, VIC, Australia; ^2^ School of Applied Systems Biology, La Trobe University, Bundoora, VIC, Australia

**Keywords:** Rhodes grass, genome sequence, genetic diversity, tetraploid, orphan crop

## Abstract

Genomic resources for grasses, especially warm-season grasses are limited despite their commercial and environmental importance. Here, we report the first annotated draft whole genome sequence for diploid Rhodes grass (*Chloris gayana*), a tropical C4 species. Generated using long read nanopore sequencing and assembled using the Flye software package, the assembled genome is 603 Mbp in size and comprises 5,233 fragments that were annotated using the GenSas pipeline. The annotated genome has 46,087 predicted genes corresponding to 92.0% of the expected genomic content present via BUSCO analysis. Gene ontology terms and repetitive elements are identified and discussed. An additional 94 individual plant genotypes originating from three diploid and two tetraploid Rhodes grass cultivars were short-read whole genome resequenced (WGR) to generate a single nucleotide polymorphism (SNP) resource for the species that can be used to elucidate inter- and intra-cultivar relationships across both ploidy levels. A total of 75,777 high quality SNPs were used to generate a phylogenetic tree, highlighting the diversity present within the cultivars which agreed with the known breeding history. Differentiation was observed between diploid and tetraploid cultivars. The WGR data were also used to provide insights into the nature and evolution of the tetraploid status of the species, with results largely agreeing with the published literature that the tetraploids are autotetraploid.

## 1 Introduction

Rhodes grass (*Chloris gayana*) is a tropical C4 species native to most of Africa that is grown across the continent as hay and permanent pasture, as well as to control erosion due to its deep rooting (Skerman and Riveros, 1990). It is a member of the genus *Chloris* that includes 56 species and sub-species, most of which are weedy types (Anderson, 1974). Rhodes grass is most closely related to *Chloris virgata*, which is more commonly known as feather fingergrass or occasionally Rhodes grass or feathertop Rhodes grass (Edwards, 2012; Rojas-Sandoval, 2016). Exported in the early 1900s, *C. gayana* is now cultivated in predominantly tropical and sub-tropical areas across the globe (Loch et al., 2004). Demonstrated by its extensive distribution in its native Africa and its establishment in non-indigenous regions, Rhodes grass has wide environmental adaptation potential, proliferating in a wide range of soil types and with cultivars capable of growing at temperatures between 8-35°C (Loch et al., 2004). Rhodes grass is a halophytic grass, capable of tolerating higher levels of soil salinity than many other C4 grasses due to its ability to partition salt ions such that photosynthesis is unaffected (Bogdan, 1969; Oi et al., 2022). Its tolerance of salt has seen the species used to restore highly degraded soils and to provide an additional source of fodder on otherwise underutilised land (Abate et al., 2021; Singh et al., 2022). Global warming-mediated temperature changes in traditionally temperate regions has increased interest in Rhodes and other C4 tropical grasses as they are considered potentially important to the future of a range of livestock industries; despite the majority of current research focusing on C3 forages (Leegood, 2013).

As a member of the PACMAD clade that includes all C4 grasses, Rhodes grass falls under the Cynodonteae tribe within the Chloridoideae subfamily (Edwards, 2012). Only four of at least 850 recognised Cynodonteae species have whole or draft genomic sequences available (*Oropetium thomaeum*, 245Mb genome; *Eleusine coracana*, 1453Mb genome; *Eleusine indica*, 584Mb genome; and *Cynodon transvaalensis*, 454Mb genome) (VanBuren et al., 2015; Hittalmani et al., 2017; Soreng et al., 2017; Zhang et al., 2019; Cui et al., 2021). While no molecular dating of the divergence of *C. gayana* from other grass species is available, it belongs to the subtribe *Eleusininae* which is known to have originated approximately 22mya and *C. gayana*’s closest known relative, *C. virgata*, is estimated to have diverged from a different *Chloris* species approximately 3.6mya (Edwards, 2012; Wang et al., 2022). Of the four Cynodonteae species with available draft genome sequences, those most closely related to the *Chloris* genus, *E. coracana* and *E. indica*, are approximately 15 million years diverged (Wang et al., 2022). The evolutionary distance from Rhodes grass to these other species makes comparative genomics challenging. Furthermore *E. coracana* and *E. indica* are both allotetraploids, which limits their application for a diploid genome assembly, and all four Cynodonteae species with genome sequence are assembled only to a scaffold level. *Setaria italica* is the most closely related species to *C. gayana* that is both a diploid and has a chromosome-level genome assembly available (Bennetzen et al., 2012).

Rhodes grass has both diploid (2n=20) and tetraploid varieties (2n=40), with some reports of triploid (2n = 30) varieties existing (Anderson, 1974; Loch et al., 2004). The tetraploid varieties are currently proposed to be autotetraploids or segmental allotetraploids based on findings from a single study (Nakagawa et al., 1987). However, the exact genetic composition of the tetraploid varieties is unknown and to date no suggestions of speciation events have been proposed (Loch et al., 2004).

Within the current known range of grass genome sizes (the smallest being *Orpetium thomaeum* at 245Mb and one of the largest being *Triticum aestivum* L. at ~17Gb), the estimated genome size of Rhodes grass is relatively compact (1C = ~293Mb) (Bennett and Smith, 1976; VanBuren et al., 2015; Shi and Ling, 2018). As an outbreeding species, it is highly heterozygous with substantial intra- and inter-cultivar variability (Loch et al., 2004; Ubi et al., 2004). This diversity gives rise to a range of beneficial traits that can be exploited by breeders. However, modern breeding tools such as genomic selection and genome editing have not yet been applied as limited research has been conducted on the species. Hence, Rhodes grass can be considered an orphan crop as it is one of the most important perennial C4 forage grasses, yet its available genomic and genetic resources are disproportionately few (Gondo et al., 2007). Currently available resources include a genetic linkage map (Ubi et al., 2004), unassembled short Illumina sequences available on GenBank from a DNA barcoding study (Gill et al., 2019) and short sequences from unpublished research. Previous research has utilised conventional anonymous molecular marker techniques such as RAPD, AFLP, ISSR and SRAP markers to examine diploid and tetraploid Rhodes grass diversity (Pérez et al., 1999; Ubi et al., 2000; Ubi et al., 2001; Ubi et al., 2003; Ribotta et al., 2019). The main conclusions of all these studies were similar; in Rhodes grass, there is a large degree of both intra- and inter-cultivar variation. The most recent study on Rhodes grass population structure and genetic diversity assessed 104 accessions using SNP and SilicoDArT markers generated with DArTseq (Negawo et al., 2021). This study examined Rhodes grass accessions in the context of their geographical origin but found no clear relationship between country of origin and genetic similarity. Consistent with the previous molecular marker studies, Rhodes grass was found to have considerable genetic diversity between accessions (Ubi et al., 2000; Ubi et al., 2001; Ubi et al., 2003). In their study, Negawo et al. (2021) attempted to align their molecular markers to reference genomes of closely related species but only a small proportion (1-5.5% of SNP markers) could be mapped to the available *Chloridoideae* genomes. They noted the number of mappable markers was significantly higher when a reference genome for that species was used, highlighting the importance of whole genome sequence resources. As their study focussed on describing a geographically diverse collection of Rhodes grass, it did not sample individual accessions deeply or provide information on ploidy status.

Due to its small genome size and genome plasticity (Nakagawa et al., 1987), *Chloris gayana* has potential as a model C4 grass species with the development of relevant underpinning genomic resources. Here, we present the first *de novo* draft reference genome assembly in the *Chloris* genus, as well as a large SNP resource developed via whole genome resequencing of a range of current commercial diploid and tetraploid cultivars of Rhodes grass. The SNP resource enables identification of within and between cultivar relationships, while the WGR data itself can be aligned to the diploid reference genome to provide insights into tetraploid Rhodes grass origins. These genomic resources will facilitate future breeding efforts and genome editing studies, as well as providing further insight in the grass phylogeny.

## 2 Materials and methods

### 2.1 Plant materials and genomic DNA extraction

Rhodes grass cv. Tolgar, line 108 (T108) is a diploid tissue culture responsive genotype selected for its high *in vitro* regeneration response and transformability, and ability to be maintained clonally by micropropagation. Young leaves of T108 from tissue culture grown, clonally propagated plants were used for DNA extraction. Approximately 200mg of leaf tissue was frozen in liquid nitrogen, ground to a fine powder, lysed in 500µL CTAB extraction buffer (100mM Tris-HCl pH 8.0, 1.4M NaCl, 20mM EDTA, 2% w/v CTAB, 2% w/v PVP-40) with 2µL (100mg/mL) RNAse and incubated for 20 minutes at 65°C. After extraction with 500µL chloroform/isoamyl (24:1) and centrifugation for 10 minutes at 16,000g, the supernatant was removed, combined with 0.7X volume of isopropanol and stood for 10 minutes at room temperature, followed by centrifugation for 10 minutes at 16,000g. The liquid phase was decanted, before 200µL of 70% (v/v) ethanol was added to the DNA pellet followed by centrifugation for 5 minutes at 13,000g. Following decanting of the ethanol, the DNA pellet was allowed to air dry before resuspension in 50µL nuclease-free water and storage at -20°C. DNA quality was assessed by Genomic DNA ScreenTape (Agilent 2200 TapeStation) according to the manufacturer’s instructions (Agilent Technologies, Santa Clara, CA, United States).

DNA was extracted from young leaf tissue of 94 Rhodes grass plants from five cultivars (Finecut, Tolgar, Endura, Mariner and Toro; **Supplementary Table 1**) from a combination of glasshouse and tissue culture grown plants using the DNeasy 96 Plant Kit (Qiagen, Hilden, Germany) as per the manufacturer’s instructions. The DNA was quantified using a Qubit 3.0 Fluorometer (Invitrogen™, Thermo Fisher Brand, CA, United States) and stored at -20°C.

### 2.2 Library preparation and genome sequencing

Enrichment of high molecular weight DNA from the CTAB-extracted samples was performed using the Size Selection Protocol from the Circulomics Short Read Eliminator Kit (PacBio, Menlo Park, CA, United States). DNA sample integrity was assessed using Genomic DNA ScreenTape (Agilent 2200 TapeStation) according to the manufacturer’s instructions (Agilent Technologies, Santa Clara, CA, United States). A genomic DNA library was constructed using the Ligation Sequencing Kit (Oxford Nanopore Technologies, United Kingdom; Genomic DNA by Ligation (SQK-LSK11)) as per the manufacturer’s instructions with a minor modification; when preparing the library for loading onto the flow cell, 51µL DNA library and 24µL loading buffer were used. The prepared DNA library was loaded onto a PromethION R9.4 flow cell and sequenced as per the manufacturer’s instructions (Oxford Nanopore Technologies, Oxon, United Kingdom).

DNeasy extracted samples were prepared for short-read sequencing using a Nextflex Rapid XP DNA-Seq Kit (Perkin Elmer, Waltham, MA, United States) as per manufacturer’s instructions with a minor modification at the Adapter Ligation step, where in-house adaptors were used instead of NextFlex barcodes. DNA quantity and length was determined using Genomic DNA ScreenTape (Agilent 2200 TapeStation) performed according to the manufacturer’s instructions (Agilent Technologies, Santa Clara, CA, United States). The samples were sequenced using an Illumina NovaSeq 6000 according to the manufacturer’s instructions (Illumina, San Diego CA, USA).

### 2.3 Genome assembly and annotation

The Oxford Nanopore Technologies (ONT) reads were assembled using the Flye (v-2.8.2, https://github.com/fenderglass/Flye) software package with the following parameters: estimated genome size set to 300Mb, minimum overlap of 10kb, the 40x longest reads for contig assembly and all reads for error correction (Kolmogorov et al., 2019). The assembled Rhodes grass genome was annotated using the GenSAS pipeline (Humann et al., 2019), which consists of multiple tools. The tools used were RepeatModeler (*de novo* repeat identification and repeat masking), followed by a BLASTn alignment to the *Setaria italica* genome (Bennetzen et al., 2012) (default settings for transcript alignments), and Augustus for gene prediction using *Zea mays* as a reference. Finally, Evidence Modeler was used to create a consensus gene set from the Augustus and BLASTn outputs, followed by PASA refinement using the *Setaria italica* genome (GenBank assembly accession: GCA_000263155.2) as a reference, and functional annotation with BLASTp (Bennetzen et al., 2012).

Strudel (Bayer et al., 2011) was used for a preliminary assembly of pseudomolecules using the *S. italica* genome as a reference for genomic locations. To prepare data for analysis in Strudel, regions of similarity between the coding sequence of Rhodes grass contigs and the *S. italica* genome were identified using BLAST. Contig locations for the subsequent list of aligned Rhodes grass genes were determined and filtered by removing contigs that aligned to more than one *S. italica* chromosome. These filtered contig lists were then concatenated with N’s to make pseudomolecules and used for alignment in Strudel.

Gene functions were identified in GenSAS using DIAMOND (protein dataset: NCBI refseq plant), after which the UniProt Retrieve/ID Mapping tool was used to identify GO annotation terms for the gene set and the quantity of genes relating to each function was determined and plotted using WEGO (Ye et al., 2006; Huang et al., 2011; Ye et al., 2018; The UniProt Consortium et al., 2022).

### 2.4 Assessment of cultivar diversity

The Illumina paired-end short read sequences were used to identify SNPs and assess sample diversity. The short reads were aligned to the draft Rhodes grass genome sequence assembly using BWA-MEM (Li and Durbin, 2009). Initial SNP discovery was performed using two software packages: GATK and SAMtools mpileup (Van der Auwera and O’Connor, 2020; Danecek et al., 2021). Identified SNP, common between the GATK and SAMtools mpileup pipelines were identified using BCFtools isec (Danecek et al., 2021). These SNP were split into diploid- and tetraploid-only sets, and each was filtered with VCFtools (minor allele frequency 0.1, maximum missing sites 0.5 and minimum sequence read depth of 4) (Danecek et al., 2011). SNP loci were then manually removed from the dataset if the genotype calls for more than 75% of the samples at a given SNP locus were genotyped as heterozygous. Finally, a SNP locus was discarded if it had both an absent homozygous allele class and the proportion of heterozygous calls in the population was greater than 60%. The filtered diploid and tetraploid lists were then compared using BCFtools isec to obtain a high confidence subset of SNP loci that overlapped between the datasets to be used as the tetraploid SNP set for building the phylogenetic tree in the subsequent steps (Danecek et al., 2021).

The tetraploid and diploid data were combined and transformed using a neighbour joining algorithm within R Statistical Software using the vcfR, NostalgiR, tidyverse and StAMPP packages (Pembleton et al., 2013; Nguyen, 2015; Knaus and Grünwald, 2017; Wickham et al., 2019; R Core Team, 2022). To enable the diploid and tetraploid samples to be compared, all samples were treated as if they were diploid and hence the allelic dosage of the tetraploid samples in the heterozygous state was not estimated. A phylogenetic tree based on these relationships was constructed in R Statistical Software using the ggplot2, ape and ggtree packages (Wickham, 2016; Paradis and Schliep, 2019; R Core Team, 2022; Yu, 2022). SnpEff was used to annotate the SNP variants and provide predictions of the effect of different SNPs (Cingolani et al., 2012). As per the SnpEff documentation, a database entry for Rhodes grass was built and the final SNP set (comprising both diploid and tetraploid individuals and containing 75,777 SNPs) was annotated.

The data presented in this study are publicly available and all genome sequence data can be found in NCBI under the BioProject ID PRJNA974075.

## 3 Results

### 3.1 Draft genome sequence assembly

The total sequence data generated by ONT long-read sequencing was 46.9Gb, an expected 156x coverage of the estimated haploid genome, with a raw read length N50 of 19.3kb. The total size of the assembled genome was 603Mb, comprising 5233 fragments with a N50 of 310kb (**Table 1**). The largest fragment was 8.8Mb. The assembled genome had a mean coverage of 76x and included 45.29% GC content and about 52% interspersed repetitive elements (**Table 2**). The predominant repeat types were retroelements and LTR elements which accounted for 31.49% and 29.44% of the repeats, respectively (**Table 2**). A total of 46,087 predicted genes were identified. An assessment of the completeness of the Flye genome assembly performed using BUSCO analysis (BUSCO 5.0.0, dataset poales_odb10) found 91.97% of the expected gene content to be present and complete, of which 71.59% were single copy and 20.38% were duplicated. The remainder comprised 2.06% of fragmented and 5.96% missing BUSCOs (**Table 1**).

**Table 1:** Summary statistics of the Rhodes grass whole genome sequence assembly and annotation.

Details	Value
Estimated genome size	2C= ~586Mb
Assembled genome size	603,261,687 bp
Number of fragments	5,233
Fragment N50	310,098 bp
Largest fragment	8,824,165 bp
Mean coverage	76x
Overall complete BUSCOs	91.97%
Complete and single-copy BUSCOs	71.59%
Complete and duplicated BUSCOs	20.38%
Fragmented BUSCOs	2.06%
Missing BUSCOs	5.96%
GC Content	45.29%
Number of predicted genes	46,087

**Table 2:** Repetitive sequence and their proportion in Rhodes grass genome.

Repeat type	Number of elements	Length occupied (bp)	Percentage of sequence
Retroelements	111,680	189,986,408	31.49
SINEs	608	148,775	0.02
LINEs	22,725	12,220,339	2.03
LTR Elements	88,347	177,617,294	29.44
DNA transposons	36,947	23,533,254	3.90
Rolling-circles	3,006	1,221,082	0.20
Unclassified	262,656	99,192,100	16.44
Total interspersed repeats		312,711,762	51.84

**Figure 1:**
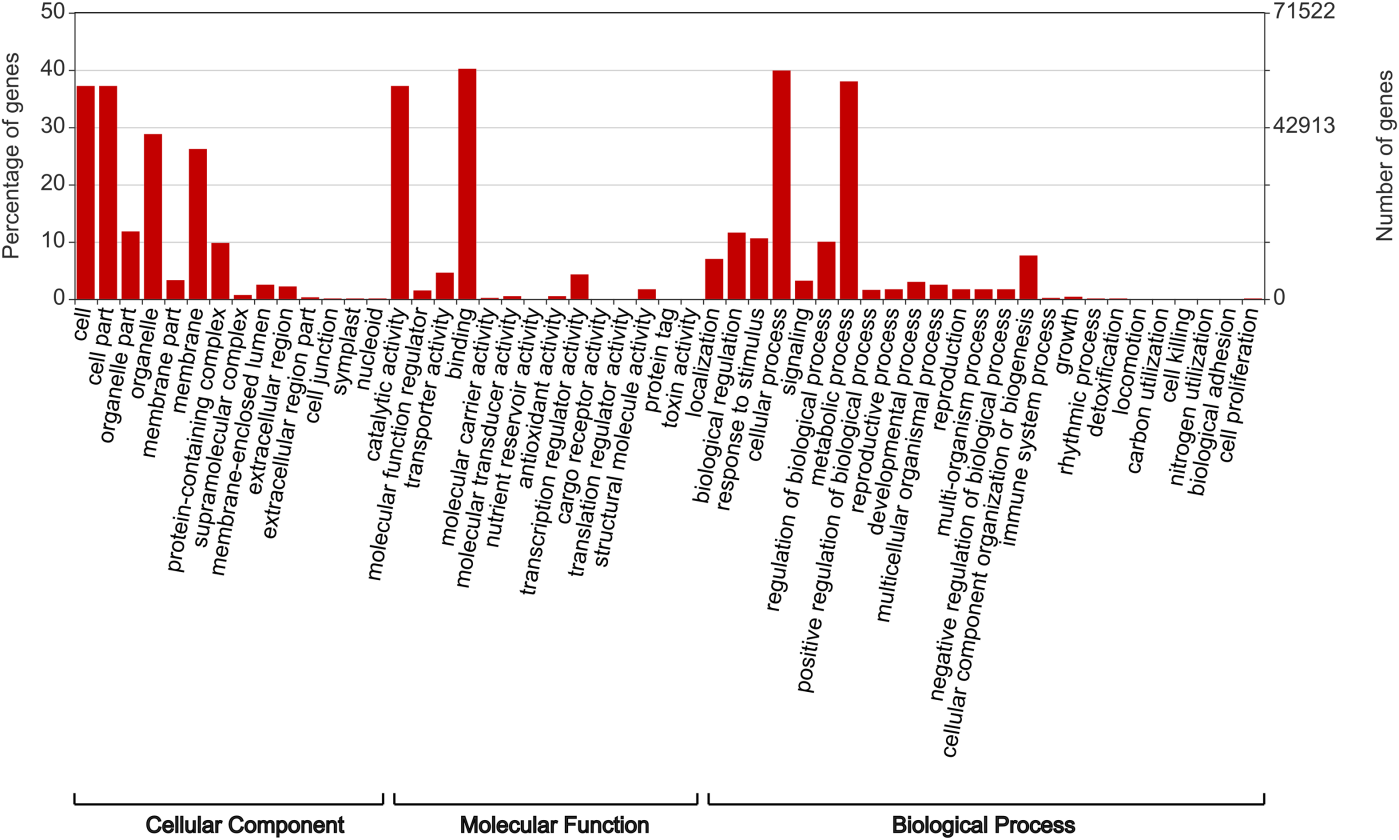
The predicted function of Rhodes grass genes as determined by DIAMOND functional analysis and plotted using WEGO.

For the complete BUSCO genes, DIAMOND analysis returned 828,107 hits due to multiple matches for each gene. Following the use of UniProt Retrieve/ID Mapping tool to remove duplicate protein entries, a total of 143,045 gene matches remained, of which 117,625 were assigned at least one GO term (**Table 3**). The assigned gene functions (**Figure 1**) split almost evenly into biological processes (31.21%), cellular components (31.52%) and molecular function (37.27%).

**Table 3:** Summary of the main predicted Rhodes grass gene functions from WEGO.

		Number of genes	Percent (%)
	Total	143,045	
	Annotated genes	117,625	82.23
GO terms	Biological process	76,326	31.21
	Cellular component	77,091	31.52
	Molecular function	91,169	37.27
	Total	244,586	

**Figure 2:**
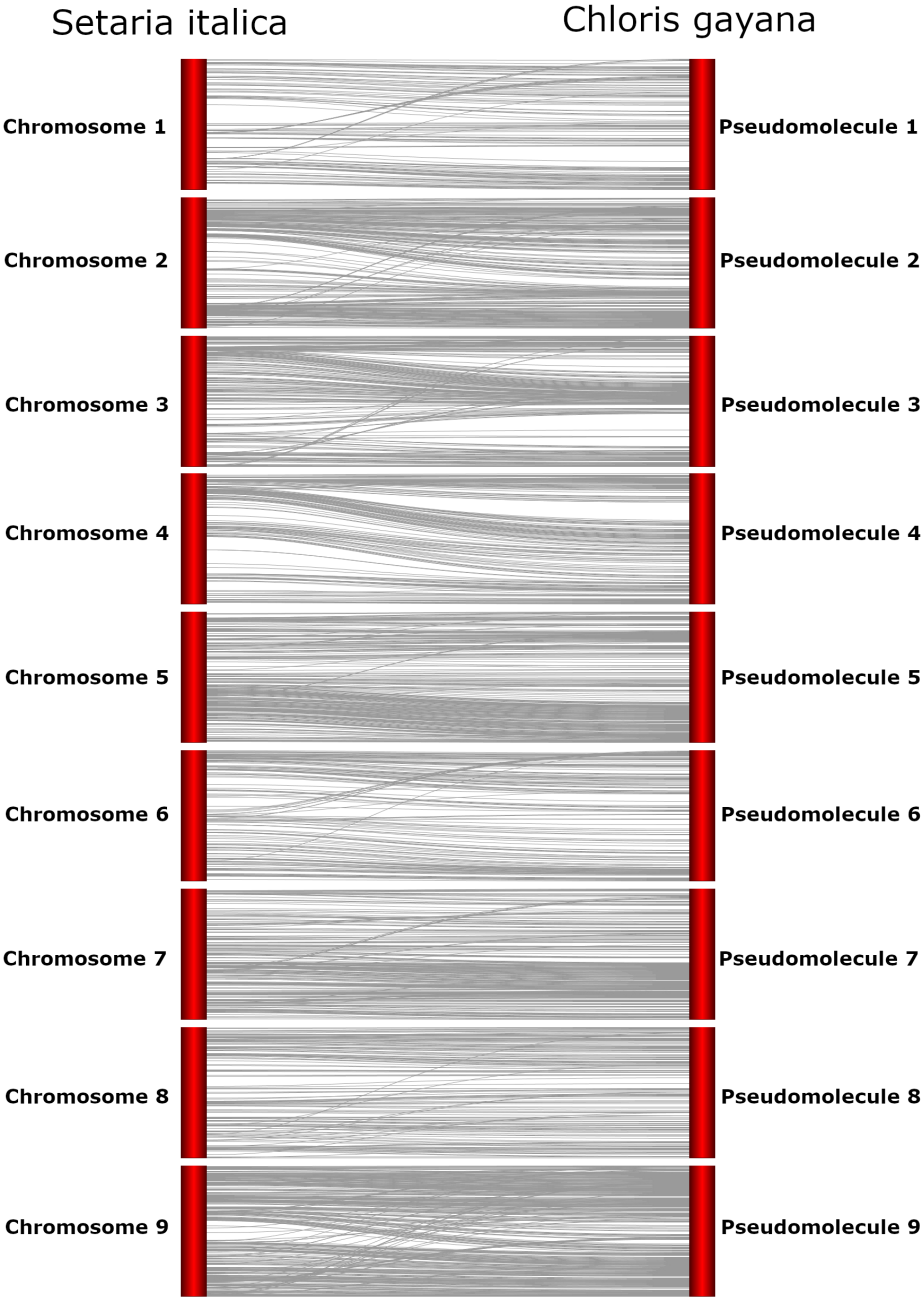
Preliminary Rhodes grass pseudomolecules aligned to *Setaria italica* genome assembly, visualised using Strudel (Bayer et al., 2011). Chromosome numbers are presented for the *Setaria italica* genome (left, in order 1-9 from the top to bottom of the image) and that numbering convention has been preserved for the *Chloris gayana* hypothetical pseudomolecules (right).

Alignment of the assembled Rhodes grass contigs to the published *Setaria italica* genome allowed the identification of an ordered set of contigs that could be considered an evaluation of genome assembly completeness. Of the 5233 assembled contigs, 1362 (~153Mb, approx. half of the haploid genome size) uniquely aligned to the nine *S. italica* chromosomes. The remaining 3871 Rhodes grass contigs were discarded as they were unaligned or had alignments to multiple *S. italica* chromosomes. The uniquely aligned Rhodes grass contigs were ordered based on *S. italica* alignment and arranged into 9 pseudochromosomes (**Figure 2**).

### 3.2 Whole genome resequencing for variant discovery

Ninety-four individual plants representing five commercial diploid and tetraploid Rhodes grass cultivars were used to generate short-read sequence data. Alignment of the resulting 1,454,838,833 reads to the draft Rhodes grass genome assembly found that the vast majority mapped with high confidence. The alignment quality did not differ between the diploid (98.02% of reads mapped, 83.87% properly paired) and tetraploid (97.63% mapped, 83.55% properly paired) datasets. The average depth of sequencing per individual was 2.34x. A total of 38,307,269 and 38,686,098 SNP were identified with GATK and mpileup, respectively, of which 26,019,620 SNP overlapped between these two genotype calling pipelines and were subjected to subsequent filtering steps. Following filtering, 75,777 diploid and 380,133 tetraploid SNP remained, of which 26,544 were common between the two sets. Attrition at each filtering step is shown in **Supplementary Table 2**.

**Figure 3:**
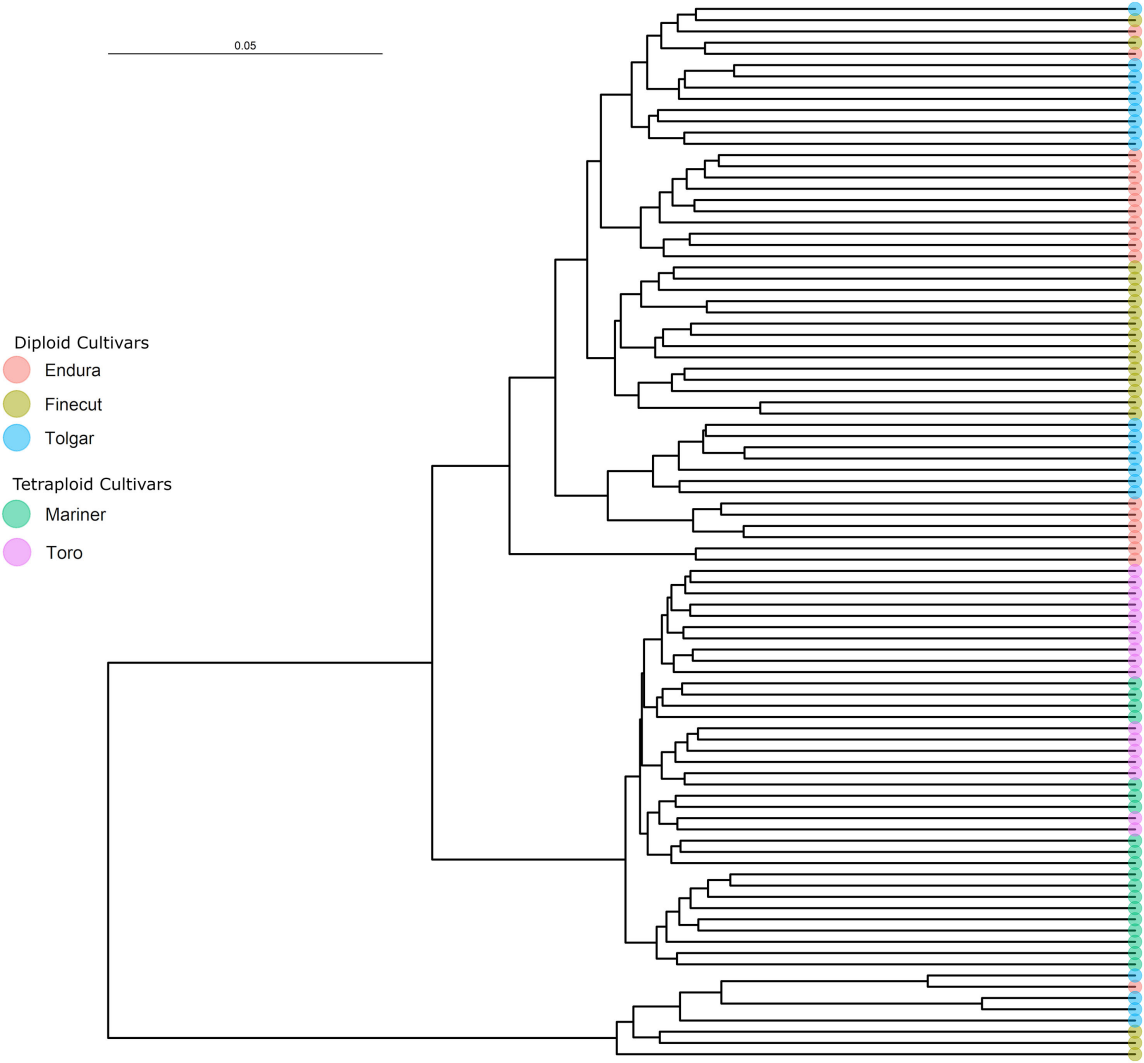
Rhodes grass phylogenetic tree based on a neighbour-joining distance matrix of filtered SNP relationships between diploid and tetraploid individuals.

The subset of SNP common between the diploid and tetraploid samples was used to assess the relationships between individuals from the five Rhode grass cultivars. A phylogenetic tree of the diploid and tetraploid samples broadly split into three branches, with one branch encompassing the tetraploids and the other two including all diploid samples (**Figure 3**). The tetraploid branch included two subgroups, one comprised of nine Mariner samples and the other of another four subgroups, of which two were solely Mariner and Toro samples containing four and ten individuals, respectively. The other two subgroups were combinations of tetraploid genotypes, altogether consisting of the remaining six Mariner and seven Toro samples. Separated by this tetraploid branch were the two clusters of diploid individuals, where the smaller diploid branch was not notably clustered by cultivar but rather consisted of four Tolgar, three Finecut and one Endura sample. The larger branch consisted of two subgroups, one of which contained four Endura and six Tolgar samples, each of which clustered according to cultivar. The remaining samples split into two groups, one containing cultivar-clustered Finecut (seven samples) and Tolgar (nine samples) individuals. The remaining branch included samples from all diploid cultivars (four Endura, nine Finecut and one Tolgar), with clustering between cultivars. Phylogenetic trees constructed for each of the diploid and tetraploid samples using the ploidy-specific SNP are shown in **Supplementary Figures 1**, **2**.

The largest grouping (43.52%) of SNP annotated by SnpEff were found in intergenic regions, followed by SNPs either 5kb upstream (21.33%) or downstream (20.06%) of a gene (**Table 4**). Synonymous (5.94%), missense (5.37%) and intron (3.52%) variants were the next largest SNP categories, with the remaining categories altogether comprising less than 1% of variant types. A summary of SNP annotations is provided as a **Supplementary file**.

**Table 4:** The type of effects predicted to be caused by SNPs in the Rhodes grass genome as determined by SnpEff (Cingolani et al., 2012).

Type	Count	Percent (%)
Downstream gene variant	26,016	20.057
Initiator codon variant	1	0.001
Intergenic region	56,451	43.520
Intron variant	4,561	3.516
Missense variant	6,959	5.365
Splice acceptor variant	10	0.008
Splice donor variant	16	0.012
Splice region variant	224	0.173
Start lost	21	0.016
Stop gained	67	0.052
Stop lost	9	0.007
Stop retained variant	3	0.002
Synonymous variant	7,705	5.940
Upstream gene variant	27,669	21.331

## 4 Discussion

An annotated draft genome sequence assembly of Rhodes grass was constructed using long-read sequencing technology and the tissue culture responsive line T108 isolated from the diploid cultivar Tolgar. At 603 Mb, the total length of the draft assembly is about the estimated diploid genome size for Rhodes grass (2C= ~586Mb) (Bennett and Smith, 1976; VanBuren et al., 2015; Shi and Ling, 2018). Due to the Flye assemblers (v-2.8.2, https://github.com/fenderglass/Flye) attempts to collapse haplotigs, the assembled genome was expected to be smaller than the actual generated assembly (603Mbp), with this larger than expected assembly potentially providing an indication of the genetic diversity of the genome. While the error rate of the ONT pore 9.4 is higher than other technologies, it is unlikely it would be sufficiently significant to cause the large assembly size of the genome. An additional hypothesis regarding the assembled data size could be seed contamination or mislabelling such that the plant sequenced to generate whole genome sequence (Tolgar 108) is tetraploid and not diploid. However, as this plant genotype was resequenced and analysed in the broader cohort of plants and subsequently shown to align more with diploid individuals (**Figure 3**), this also may be unlikely. Comprising 5,233 contigs with a N50 of 310kb and 46,087 predicted genes (including 91.97% of expected BUSCO genes, of which 71.59% are single copy), the quality and completeness of the draft genome assembly is comparable to that of other draft orphan crop genomes; e.g. *Digitaria exilis*- 3329 contigs, BUSCO: 98.1% (Wang et al., 2021). As generated by EvidenceModeler from the consensus of the Augustus gene predictions and the *S. italica* BLASTn alignment, the predicted gene number for Rhodes grass (46,078) was in a similar range of gene number compared to closely related species *E. coracana* (57,180 predicted genes (Hatakeyama et al., 2018)), *S. italica* (24,000-29,000 predicted genes (Bennetzen et al., 2012)) and *L. perenne* (38,868 predicted genes (Frei et al., 2021)). The accuracy of the Rhodes grass predicted gene set could be improved by creating transcriptome resources for training prediction models, however this was outside the scope of this study.

Among commercial cultivations of Rhodes grass, cultivars descended from Katambora are common. Hierarchical clustering of 104 Rhodes grass accessions by Negawo et al. (2021) showed the presence of two main subpopulations, with Katambora clustering within the larger group. As Tolgar is a descendent of Katambora, the draft genome sequence generated is expected to be useful as a representative for diploid Rhodes grass and broadly applicable for a majority of Rhodes grass cultivars.

The reference genome for *Setaria italica*, that is assembled to chromosome level, was used to order the assembled draft Rhodes grass genome contigs, based on synteny and visualised using Strudel (Bayer et al., 2011; Bennetzen et al., 2012). *Setaria italica* is the most closely related species to Rhodes grass with an available whole genome sequence and has a similar number of chromosomes to diploid Rhodes grass (*S. italica* 2n = 18; diploid *C. gayana* 2n = 20). The *S. italica* subfamily Panicoideae is estimated to have diverged from the other PACMAD subfamilies about 20mya (Cotton et al., 2015). The comparative alignment and syntenic comparison provides an indication of genome assembly completeness, and a preliminary indication of how the Rhodes grass contigs may be ordered at a chromosome level. The contig ordering and alignment from this approach has been provided for reference only and has not been used for any further analysis. The availability of a Rhodes grass high-density genetic linkage or optical map anchored to chromosomes would allow for the contigs to be more accurately assembled into pseudochromosomes (You et al., 2018). However, to date molecular genetic studies for Rhodes grass have generally focussed only on assessing cultivar diversity (Pérez et al., 1999; Ubi et al., 2000; Ubi et al., 2001; Ubi et al., 2003; Ribotta et al., 2019). To our knowledge, only one genetic linkage map has been constructed for Rhodes grass based on 25 restriction fragment length polymorphisms (RFLPs) and augmented with amplified fragment length polymorphisms (AFLP) markers (Ubi et al., 2004). The density of this linkage map is insufficient for anchoring and ordering the draft Rhodes grass genome assembly.

It is known from available breeding information (**Supplementary Table 1**) that the diploid cultivars Finecut, Tolgar and Endura are all descendants of Katambora, a landrace derived from seed collected along the Northern Rhodesian bank of the Zambesi River in Zimbabwe (CSIRO, 1972b). Hence, the overlapped grouping of the Rhodes grass diploid cultivars was expected due to their shared ancestry and may also reflect the limited selection and outcrossing nature of its breeding system (**Figure 3**). In general, the diploid samples clustered according to the cultivar from which they were derived, although there was some overlap and mixing most likely due to their shared ancestry from cv. Katambora. The tetraploid cultivars Toro and Mariner clustered into their own group, separate from the diploid samples, with some mixing between cultivars. Despite this, these cultivars have not been reported to share close genetic or geographic origin. Mariner and Toro are descended from cultivars Samford and Callide, respectively (Plant Breeders Rights, 2022; Barenbrug, 2022a; Barenbrug, 2022b). Callide originates from what is now Tanzania, while Samford was derived from ecotypes grown in Sierra Leone that arose from a prior introduction from Kenya (CSIRO, 1972a; CSIRO, 1972c). The tetraploids cluster away from the diploids in our phylogeny, as they are likely derived from a speciation event and therefore have more closely linked genetic ancestry. These three parental cultivars, Katambora, Callide, and Samford, were shown by Negawo et al. (2021) to cluster together when evaluated against other Rhodes grass cultivars, indicating a degree of similarity despite differences in geographic origin and ploidy. The pedigree of the cultivars likely contributes to the overlapping and integrated clades within the Rhodes grass phylogenetic tree. It is also possible that some overlap of individuals among cultivars results from inadvertent seed mixing or interbreeding in the field. The SNP lists used to construct the phylogeny were filtered with strict parameters, which likely resulted in the exclusion of some genuine SNPs and rare alleles. However, as these data are the first of their kind for Rhodes grass we have chosen to provide the most conservative highest quality SNP set as a foundation for future research, also considering the modest average sequence coverage and samples per cultivar that the study generated.

Previous cytologic work has suggested that tetraploid Rhodes grass is likely to be an autotetraploid or segmental allotetraploid due to the observed presence of multiple multivalent formations during meiosis (Nakagawa et al., 1987). Autotetraploids have two duplicated copies of the genome originating from a single ancestor, while segmental allopolyploids originate from doubled diploid hybrids of closely related species with chromosome pairing generally favoured between common ancestors (Sybenga, 1996). Given there was little difference (0.39%) in the proportion of diploid and tetraploid sample reads that were mapped to the diploid reference genome, our results suggest the second sub-genome of the tetraploid cultivars is sufficiently similar to the diploid reference to correctly align. The consistency of alignment suggests that the tetraploids originated from a self-genome replication event consistent of an autotetraploid. If the tetraploids were segmental allotetraploids, as has been previously suggested, a considerable difference in the alignment statistics might be expected (Nakagawa et al., 1987; Loch et al., 2004). A much higher frequency of heterozygous SNP genotype calls would also be expected for the tetraploid samples when their sequence reads were mapped to the diploid genome assembly due to sequence divergence between some chromosomes of one genome and those of the other genome in the tetraploid samples. Consistent with previous cytological studies which showed that tetraploid Rhodes grass forms multivalents during meiosis, our results allow us to rule out the possibility that the tetraploids are allotetraploids (two distinct genomes brought together through hybridisation between two species followed by doubling of the chromosomes of the resulting hybrid). If allotetraploids, sequence reads from the second unrepresented sub-genome would be expected to misalign with the diploid Rhodes grass genome assembly, resulting in a high frequency of heterozygous SNP genotype calls which was not observed. Further, a proportion of the SNP from the tetraploid samples would be expected to possess a nucleotide variant not observed among SNP discovered in the diploid samples. Hence, our results suggest that tetraploid Rhodes grass is an autopolyploid.

This understanding of Rhodes grass as an autopolyploid can facilitate breeding and genome editing for improved forage qualities. As the chromosome sets in tetraploid Rhodes are homologous, polysomic inheritance can occur with recombination of alleles possible between sub genomes, allowing for inheritance of desirable traits (Klie et al., 2014). Genome editing polyploids can be complicated due to circumstances including high heterozygosity, presence of homoeologous alleles and repetitive DNA. Nevertheless, genome editing of several polyploid species has been achieved with various site-directed nuclease (SDN) technologies, such as Talens and CRISPR-Cas9 (Schaart et al., 2021). Previous work on editing autotetraploid crops has been facilitated by the presence of the whole genome sequence of the autotetraploid, the comparison of autotetraploid short read sequence to a diploid genome, or by characterising specific target regions then looking for genetic variation between targets (Johansen et al., 2019; Chen et al., 2020; Sevestre et al., 2020). By generating short read sequence and SNP data for Rhodes grass tetraploids, as well as a diploid whole genome sequence, our work could facilitate future breeding and genome editing of tetraploid Rhodes grass.

The draft genome sequence generated here enables transcriptome studies on the variation of important traits in Rhodes grass, like salt tolerance and resilience to varying growth temperatures (Bogdan, 1969; Loch et al., 2004; Oi et al., 2022). Additionally, the SNP annotation data gives an insight into variations in the sequence that may impact gene function between individuals and can better explain varying phenotypes between individuals. These data will be of significant value for the research community that is gaining increased interest in this species as it will enable a wide range of molecular breeding tools as well as an array of biotechnology approaches to now be possible.

## 5 Conclusion

The draft diploid Rhodes grass genome assembly presented here represents the second draft genome assembly among Cynodonteae forages and first within the *Chloris* genus. It provides an important resource to underpin the genetic improvement of important breeding traits such as forage digestibility. Our results provide supporting evidence that is consistent with tetraploid Rhodes grass having evolved through an autopolyploid process. The genomic resources generated can be used to facilitate genomic breeding approaches and genome editing, and provide the opportunity to use Rhodes grass as a model C4 forage species for functional genomic studies. Rhodes grass is a resilient warm season forage crop, which would benefit from genetic improvement of its forage quality since it has been identified as a potential future forage species for dairy and livestock industries in regions where global warming is expected to reduce temperate pasture quality and productivity.

## Data availability statement

The datasets presented in this study can be found in online repositories. The names of the repository/repositories and accession number(s) can be found below: Bioproject accession number: PRJNA974075.

## Author contributions

The authors contributed to the paper as follows, study conception; NC, MH, KM-R, and DI: data collection; KM-R: data analysis and interpretation; KM-R: paper drafting; KM-R: paper revision and editing; KM-R, NC, DI, and MH. All authors contributed to the article and approved the submitted version.

## Supplementary material

Supplementary Figure 1 Rhodes grass phylogenetic tree based on a neighbour-joining distance matrix of filtered SNP relationships between diploid individuals

Supplementary Figure 2 Rhodes grass phylogenetic tree based on a neighbour-joining distance matrix of filtered SNP relationships between tetraploid individuals
