# Supplementary figures and images for: Development of genomic resources for Rhodes grass (Chloris gayana), draft genome and annotated variant discovery
Source: Front Plant Sci. 2023 Sep 4;14:1239290. doi: 10.3389/fpls.2023.1239290 (PMC10507473; doi:10.3389/fpls.2023.1239290)

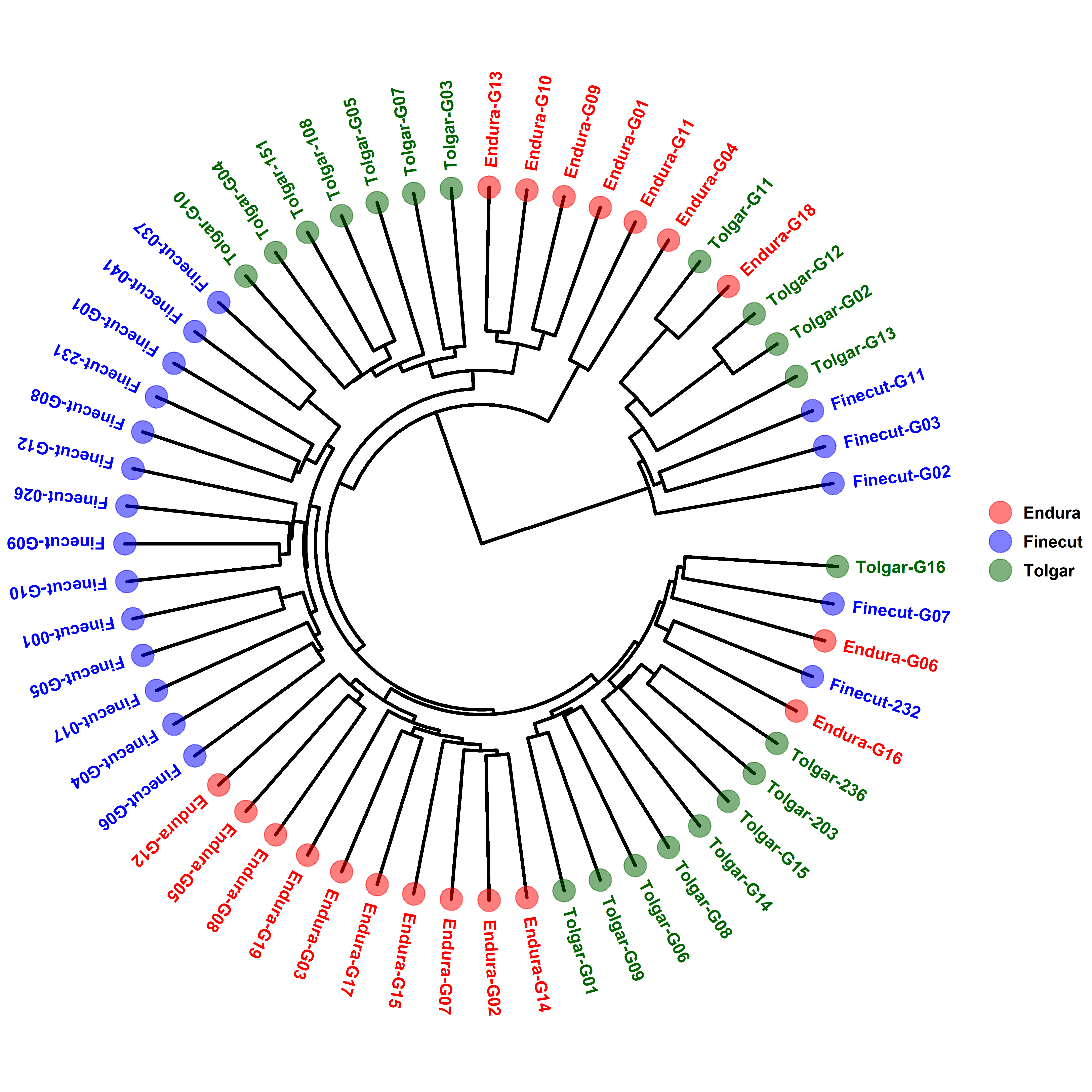

Supplement: Supplementary Figure 1 — Rhodes grass phylogenetic tree based on a neighbour-joining distance matrix of filtered SNP relationships between diploid individuals [file Image_1.jpeg]

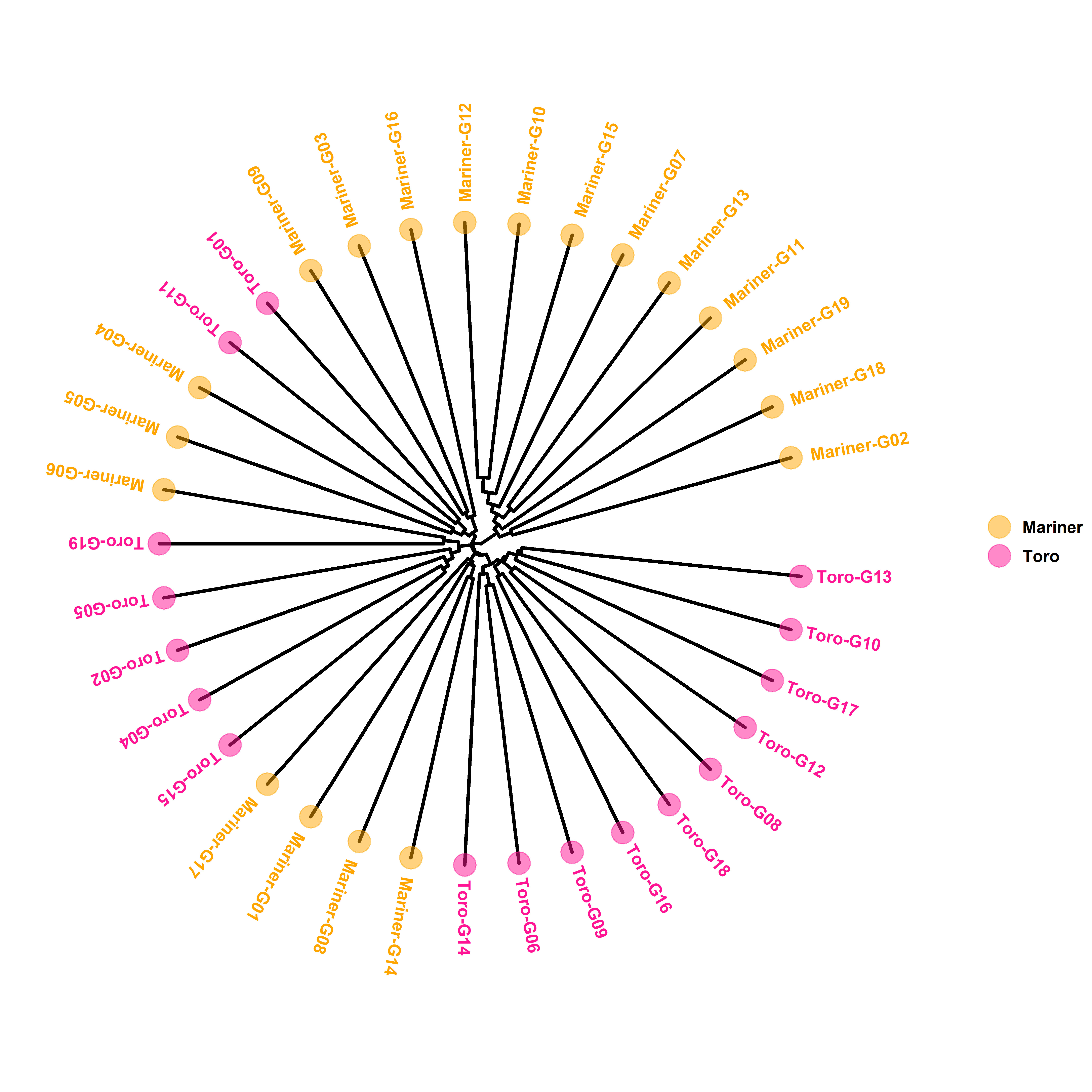

Supplement: Supplementary Figure 2 — Rhodes grass phylogenetic tree based on a neighbour-joining distance matrix of filtered SNP relationships between tetraploid individuals [file Image_2.jpeg]
