# Supplementary material for: Development of genomic resources for Rhodes grass (Chloris gayana), draft genome and annotated variant discovery
Source: Front Plant Sci. 2023 Sep 4;14:1239290. doi: 10.3389/fpls.2023.1239290 (PMC10507473; doi:10.3389/fpls.2023.1239290)
Supplement: Supplementary file 3 [file Table_1.docx]

Supplementary Table 1: Summary of Rhodes grass cultivar chromosome number, ancestral cultivar, and breeding information

| Cultivar: | Ancestral cultivar: | Number plants sampled for short read sequencing | | Ploidy: | Breeding information: |
| --- | --- | --- | --- | --- | --- |
| Endura | Tolgar | | 19 | Diploid (2n=20) | Selected from cv. Tolgar (Barenbrug, 2022b) |
| Finecut | Katambora | | 19 | Diploid (2n=20) | “Derived from one cycle of selection based on 10 clones selected from spaced plants representing two distinctly different Katambora populations” (Plant Breeders Rights, 2022) |
| Tolgar | Katambora | | 20 | Diploid (2n=20) | “Katambora type” (Barenbrug, 2022b) |
| Mariner | Samford | | 19 | Tetraploid (2n=40) | “Mass phenotypic selection was applied to four successive generations of seedlings derived from ‘Samford’ Rhodesgrass… ‘Mariner’ is a synthetic cultivar derived from the final 12 plants selected from the F4 breeding generation” (Plant Breeders Rights, 2022) |
| Toro | Callide | | 17 | Tetraploid (2n=40) | “Mass phenotypic selection was applied to four successive generations of seedlings derived from ‘Callide’ Rhodes grass… ‘Toro’ is a synthetic cultivar derived from the final 13 plants selected from the F4 breeding generation” (Plant Breeders Rights, 2022) |

Supplementary Table 2 Diploid and tetraploid filtering statistics based on genotype calls

|  | Number diploid SNPs | Percent of total unfiltered diploid SNPs (%) | Number tetraploid SNPs | Percent of total unfiltered tetraploid SNPs (%) |
| --- | --- | --- | --- | --- |
| Total number unfiltered SNPs | 100,392 |  | 411,762 |  |
| SNP sites with ≥75% heterozygosity | 14,724 | 14.67 | 17,823 | 4.33 |
| Absent homozygous allele and ≥60% heterozygosity | 9,891 | 9.85 | 13,806 | 3.35 |
| Final number of filtered SNP | 75,777 | 75.48 | 380,133 | 92.32 |
